# Significantly Reduced Genoprevalence of Vaccine-Type HPV-16/18 Infections among Vaccinated Compared to Non-Vaccinated Young Women 5.5 Years after a Bivalent HPV-16/18 Vaccine (Cervarix®) Pilot Project in Uganda

**DOI:** 10.1371/journal.pone.0160099

**Published:** 2016-08-02

**Authors:** Edward Kumakech, Vanja Berggren, Henry Wabinga, Gabriella Lillsunde-Larsson, Gisela Helenius, Malin Kaliff, Mats Karlsson, Samuel Kirimunda, Caroline Musubika, Sören Andersson

**Affiliations:** 1 School of Health and Medical Sciences, Örebro University, Örebro, Sweden; 2 Department of Pathology, School of Biomedical Sciences, Makerere University College of Health Sciences, Kampala, Uganda; 3 Faculty of Medicine, Lund University, Lund, Sweden; 4 Department of Public Health (Global Health/IHCAR), Karolinska Institute, Stockholm, Sweden; 5 Department of Laboratory Medicine, Faculty of Medicine and Health, Örebro University Hospital and Örebro University, Örebro, Sweden; Rudjer Boskovic Institute, CROATIA

## Abstract

The objective of this study was to determine the prevalence and some predictors for vaccine and non-vaccine types of HPV infections among bivalent HPV vaccinated and non-vaccinated young women in Uganda. This was a comparative cross sectional study 5.5 years after a bivalent HPV 16/18 vaccination (Cervarix®, GlaxoSmithKline, Belgium) pilot project in western Uganda. Cervical swabs were collected between July 2014-August 2014 and analyzed with a HPV genotyping test, CLART^®^ HPV2 assay (Genomica, Madrid Spain) which is based on PCR followed by microarray for determination of genotype. Blood samples were also tested for HIV and syphilis infections as well as CD4 and CD8 lymphocyte levels. The age range of the participants was 15–24 years and mean age was 18.6(SD 1.4). Vaccine-type HPV-16/18 strains were significantly less prevalent among vaccinated women compared to non-vaccinated women (0.5% vs 5.6%, p 0.006, OR 95% CI 0.08(0.01–0.64). At type-specific level, significant difference was observed for HPV16 only. Other STIs (HIV/syphilis) were important risk factors for HPV infections including both vaccine types and non-vaccine types. In addition, for non-vaccine HPV types, living in an urban area, having a low BMI, low CD4 count and having had a high number of life time sexual partners were also significant risk factors. Our data concurs with the existing literature from other parts of the world regarding the effectiveness of bivalent HPV-16/18 vaccine in reducing the prevalence of HPV infections particularly vaccine HPV- 16/18 strains among vaccinated women. This study reinforces the recommendation to vaccinate young girls before sexual debut and integrate other STI particularly HIV and syphilis interventions into HPV vaccination packages.

## Introduction

Cervical cancer (CC) is the third commonest cancer among women globally, with an estimated 527,624 new cases and 265,672 deaths in 2012 [1]. Approximately 87% of the CC deaths occur in low-income countries. In East Africa, CC is the most prevalent cancer and the leading cause of cancer-related deaths among women [1]. In Uganda a recent data from Kampala Cancer Registry indicates that the age-standardized incidence for CC rose from a rate of 44.7 per 100,000 women during the period 1996–2000 to 50.2 per 100,000 women in the period 2006–2010 [2]. Although the most recent data from Globocan 2012 put Uganda’s age-standardized incidence rate for CC at 44.4 per 100,000 women in 2012, it remains one of the highest in the world [1].

Persistent infection with sexually transmittable high-risk Human papillomavirus (HPV) is a necessary cause of cervical cancer [3]. Several mucosal HPV types including HPV 16, 18, 31, 33, 35, 39, 45, 51, 52, 56, 58 and 59 have been classified as carcinogenic Group 1 or high-risk (HR) strains because of their association with CC [4–8]. Notably, 70% of cervical cancer cases were attributable to HPV-16 and18 strains alone [9], making them excellent targets for prophylactic vaccines. Mucosal HPV type 68 was classified as possibly carcinogenic (Group 2A) while additional mucosal HPV types including HPV 26, 30, 53, 66, 67, 69, 70, 82 and 85 were classified as probably carcinogenic (Group 2B) based on their phylogenetic relatedness to Group 1 types [5–8]. Other mucosal HPV types including HPV 6 and 11 were classified as low-risk (LR) because of their association with benign genital warts [10].

Uganda also has a high prevalence of HPV infections among women with normal cervix as well as invasive cervical cancer [11–12]. More so, among young women <25 years, HPV genoprevalence range 39.8%–74.6% compared to women aged > 25 years in Uganda [13–14] making young women <25 years the appropriate age group to target with interventions aimed at primary prevention of cervical cancer through prophylactic HPV vaccines. Pre-HPV vaccine introduction studies in Uganda [14, 15–16] summarized in Table 1 below shows that HPV genotypes 16, 18, 31, 33, 35, 39, 45, 51, 52, 56, 58, 59, 68/73, 6, 11, 34, 40, 40, 42, 43, 44, 53, 54, 66, 70 and 74 were prevalent and also the persistent genotypes in cervical cancer trajectory from young women aged <25 years, young primiparious women aged <25 years, middle age women (average age of 41 years) with normal cervical cytology and old women (average age of 44 years) with invasive cervical cancer (ICC).

**Table 1:** Prevalence of HPV genotypes among various categories of Ugandan women in pre-HPV vaccine introduction period 2008–2011.

HPV genotypes	Young aged <25 year old women (n = 1275) from Banura et al 2010 study %	Young aged <25 year old primiparous women (n = 987) from Banura et al 2010 study %	Middle aged 41 year old women with normal cervical cytology (n = 309) from Odida et al 2011 study %	Old aged 44 year old women with invasive cervical cancer (n = 239) from Odida et al 2011 study %
High risk genotypes				
16	11.1	8.4	3.2	34.9
18	12.4	5.8	2.6	13.5
31	6.3	5.1	1.0	0.7
33	10.8	5.0	1.6	1.4
35	5.8	5.3	2.3	3.5
39	4.7	3.9	0.3	1.0
45	2.9	3.3	0	6.2
51	14.2	8.7	2.6	0.7
52	12.9	12.1	4.2	2.1
56	8.4	5.5	1.3	0.7
58	2.1	4.0	1.0	0.7
59	2.1	1.7	0.6	0.3
68/73	5.3	5.8	2.3	2.1
Low risk genotypes				
6	19.5	5.5	7.8	1.7
11	14.5	3.2	1.0	0.3
34	0	0.5	0.6	0
40	4.5	1.6	0.3	0
42	0.5	0.2	0.3	0
43	5.0	1.4	0	0.3
44	1.6	1.6	1.3	0
53	3.2	2.7	1.3	0
54	2.4	2.5	1.0	0.3
66	5.3	6.5	1.6	0
70	2.9	2.5	1.0	0.3
74	0.5	1.2	1.3	0.7

% is percent. HPV prevalence summarized in the Table 1 above are based on Banura et al 2010 [15] and Odida et al 2011 [14] pre-vaccine introduction studies among Ugandan women.

To address that, the Bivalent HPV16/18 ASO4-adjuvanted vaccine (Cervarix®, GlaxoSmithKline Biologicals, Belgium) was introduced in Uganda in 2008 for young girls as a primary prevention strategy for cervical cancer. In 2008 alone, over 3000 young girls aged 10–18 years who were in primary school grade 5 (P5) were given the Bivalent HPV16/18 vaccine as per the recommended schedule of 0, 1 and 6 months and a dose 3 vaccination coverage of over 95% was achieved [17]. In order to be able to follow up and determine the impact of HPV immunization, HPV vaccination registers were set up in Uganda as part of the HPV vaccination programme. The key variables in the register were the name, date of birth, address, parents name, dates and doses of HPV vaccine received by the girls. This was in addition to the school registers of all girls including the non-vaccinated girls.

Bivalent HPV immunization has been shown to reduce the genoprevalence of HPV types 16 and 18 in the vaccine trials [18–21] and post immunization surveillance studies in Germany, Scotland and England [22–24] have confirmed this. To our knowledge, such assessments of the bivalent HPV-16/18 (Cervarix®) vaccination impact in terms of impact on the HPV genoprevalence among young women soon after sexual debut has not yet been conducted in any developing country in Africa. And yet it is important to also conduct it among young women in developing countries in Africa such as Uganda because of the prevalent other sexually transmitted infections (STIs) such as HIV and syphilis which have the potential to undermine the protective effects of the HPV vaccination.

We therefore aimed to compare the unvaccinated and vaccinated 15–24 year old women 5.5 years after the bivalent HPV16/18 vaccination (Cervarix®, GlaxoSmithKline, Belgium) pilot project regarding the genoprevalence and predictors for vaccine and non-vaccine types of HPV infections to provide information on the early impact of bivalent HPV vaccination in Uganda. The findings can inform decisions on groups of girls that should be the primary target for HPV vaccination and catch up or supplementary vaccination campaigns.

## Materials and Methods

### Research Ethics Statement

The ethical approval (number SBS HDREEC-131) for the study was obtained from the Institutional Review Board (IRB) of School of Biomedical Sciences, Makerere University College of Health Sciences, Uganda. The same IRB provided approval for the consent procedure employed for participants under the age of 18 years. Additional research clearance was obtained from Uganda National Council for Science and Technology (UNCST). Informed consents were obtained from each participant aged 18 years and above. For participants below the age of 18 years, assent was obtained from them plus a proxy informed consent from their Teachers because they were self- supporting in boarding schools, not living with their parents at home. These put them in the category of emancipated minors who should be given prompt opportunity to benefit from the medical tests and treatment provided as part of the research. The participants were provided transportation to and from the health facility for sample collection. At the health facility, participants were served a soft drink and were provided the opportunity to receive healthcare services for free.

### Study Design and area

This was a comparative cross sectional study [25] conducted in Ibanda district, Uganda. Ibanda district is situated in rural southwestern Uganda. The district was selected for the study because it was one of the first districts in Uganda where pilot bivalent HPV-16/18 vaccination of young girls was implemented in 2008 through 2009.

### Study Population

The study population was women aged 15–24 years which comprised of bivalent HPV-16/18 vaccinated and non-vaccinated groups of girls. The vaccinated group was selected strictly from the 2008 cohort of bivalent HPV-16/18 vaccination. The total population eligible for the study was 3,459 girls vaccinated in 2008. At the time of vaccination in 2008, the average age of the vaccinated girls was 13 years and range was 9–18 years [17]. Girls were included in the vaccinated group of the study if they were sexually active and by 2008 were in primary school class five (P5) in Ibanda district. They should in addition be fully vaccinated with 3 doses of Bivalent HPV16/18 vaccine (vaccinated group). For the unvaccinated group of the study, girls were included if they were sexually active and by 2008 were in primary school class six (P6) and primary school class seven (P7) in Ibanda district but were not vaccinated with Bivalent HPV16/18 vaccine (non-vaccinated group). HPV partially vaccinated girls were excluded from the study because they were very few as the HPV vaccination coverage in the area in 2008 was above 95% [17]. Sexually naïve women from both the vaccinated and unvaccinated groups were excluded from the study because by being sexually naïve they stand no chance of acquiring any sexually transmissible cervical HPV infection and are therefore not eligible members of young women population at risk of sexually acquired cervical HPV infections on which inferences from the study are drawn. Apart from the above sexual activity criterion, there were no other exclusion criteria by vaccination groups.

### Sample Size Calculation

The sample size was determined by power analysis calculation [26]. The calculation for sample size for comparing proportions which makes use of the Normal approximation to the Binomial distribution was used because the primary outcome variable was categorical (i.e. HPV infection status). The genoprevalence of vaccine HPV-16 or 18 infections in the non-vaccinated groups of girls was assumed to be 10.7% based on a previous study conducted in Uganda [13]. The genoprevalence of vaccine HPV 18 or 16 infections in the vaccinated group that would represent an important improvement was assumed to be 1%. The proportions compared were thus 0.107 and 0.01. A sample size of 376 was required at a power of 85% and 5% significance level. This was adjusted to 492 participants (i.e. 241vaccinated and another 241 non-vaccinated girls) to cater for potential loss to follow up.

### Participant’s recruitment and sampling

**Fig 1:**
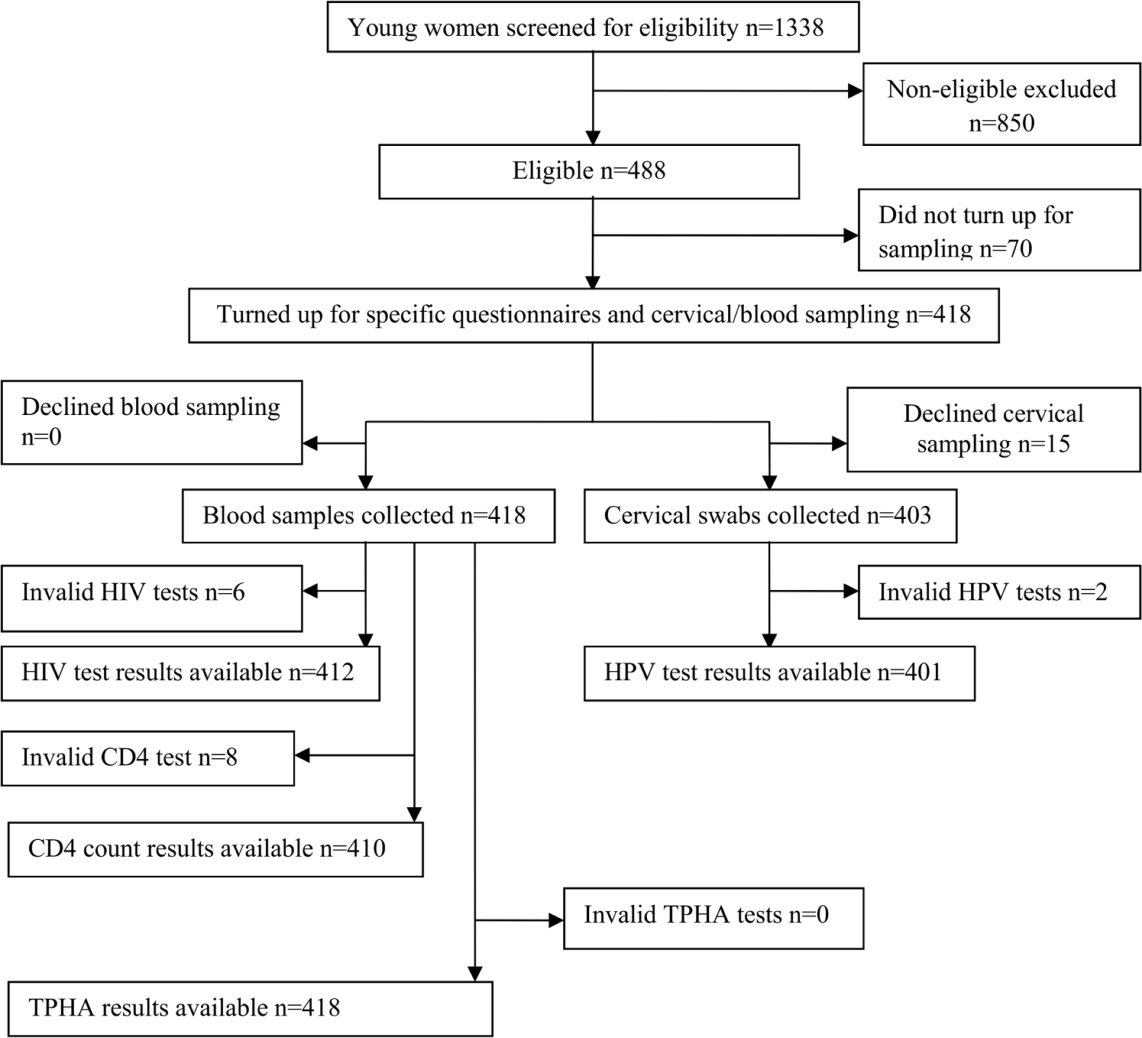
Participants flow and procedures in the study. n is the number of participants; CD4 is CD4+ T cells and TPHA is *Treponema pallidum* Hemagglutination assay, the test for syphilis.

From July 2014 –August 2014, we performed a multi-stage sampling procedure to select the study participants and collect data. We first developed a total list of senior secondary schools where the 2008 cohort of vaccinated girls and their non-vaccinated counterparts were expected to be studying at the time of the study. All the schools were visited by the research team and girls in senior secondary three (S3) to senior secondary six (S6) classes were approached, informed about the study, consented and screened for eligibility. The eligible girls were given appointment to visit a designated clinic at a health facility within the locality for sample collection. On the clinic appointment day, vehicles were sent to transport the girls to and from the health facility in company of their teachers. Fig 1 is the flow chart of the participants within the study.

### Sample collection

At the health facility, samples and data were collected from consecutive girls reaching the clinic until the required sample size was reached. The procedures performed at the clinic included re-screening for eligibility, assignment to study group using the 2008 HPV vaccination register.

### Demographic characteristic and sexual history

An interviewer-administered questionnaire was used to obtain data about the participant’s age, address, education level, age at sexual debut, number of sexual partners in the past 3 months, number of sexual partners in the past 1 year, number of sexual partners in a lifetime and condom use level.

### Weight and height

Weight was measured using a standard weight scale. Height was also measured using a standard height scale. BMI was calculated using the formula Kg/m^2^. The resultant BMI was categorized into underweight (BMI<18.5), normal weight (BMI 18.5–24.9), overweight (BMI 25.0–30.0) and obese (BMI >30.0) according to WHO criteria [27]. The personnel conducting weight and height measurements were experienced nurses who had been trained and were supervised during the entire procedure by the first author.

### Blood sampling

Venous whole blood samples were drawn from the cubical vein of each participant using 8 mL heparinized or EDTA vacutainer tubes. The blood draws were performed by 2 experienced laboratory technicians.

### HIV testing

HIV-1/2 testing was performed using HIV-1/2 Rapid Diagnostic Tests (RDTs) in accordance with Uganda national algorithm, which is based on WHO recommendations [28]. The testing began with Alere Determine® HIV-1/2 RDT (Alere Medical Co. Ltd US). If the sample was not reactive, it was considered HIV-negative. Otherwise, the Statpak HIV-1/2 RDT (Chembio Diagnostics Syst US) was used to confirm HIV positivity. In case of disagreement between the 2 tests, a tie-breaker test, the Uni-Gold™ Recombigen® HIV-1/2 RDT (Trinity Biotech PLC US), was used. For quality assurance purpose, all HIV-positive results were subsequently confirmed by EIA (Architect HIV ab/ag Combo, Abbott Laboratories, Abbot Park, IL, USA) at Örebro University Hospital, Sweden. HIV positive cases were linked to the Antiretroviral Therapy (ART) clinic available at the health facility where the HIV tests were performed.

### CD4 count

Measurement of CD4 lymphocyte counts was carried out using BD FacsCount® (Immunocytometry Systems; Becton Dickinson, Franklin Lakes, NJ, USA) at the Kiwoko Health Centre IV laboratory, Ibanda Uganda. The laboratory is designated clinical laboratory for Ibanda district and follows national standards for quality control. The BD FacsCount® protocol used for CD4 estimation is internationally acceptable, robust and known to have a low average coefficient of variation [29]. Total lymphocyte count (TLC) and haemoglobin (Hb) were determined by automated blood analyser (ABX micros 60; ABX Diagnostics, Montpellier, France). For TLC, a cut-off of 1200 cells/μl was used, as this is recommended as a substitute for CD4 counts when the latter is unavailable and HIV-related symptoms exist [29].

### Syphilis testing

Part of the venous blood collected for HIV testing and CD4 estimation was used for the Syphilis testing. Serum obtained from the venous blood at the study site was kept and transported in liquid nitrogen to Immunology Laboratory at Makerere University College of Health Sciences where it was immediately transferred to minus 80°C freezer until testing. The liquid nitrogen tanks ensured cold chain for samples during transportation from fieldwork site to the Laboratory. All the Syphilis tests were performed by an independent Laboratory Technologist (co-author WM) at Immunology Laboratory of Makerere University College of Health Sciences, Kampala, Uganda who was not part of the blood draw team. The Syphilis tests were performed using a commercially available kit SYPHILIS TPHA liquid test (Human Gesellschaft for Biochemica and Diagnostic MBH, Wiesbaden, Germany). The tests were performed according to the manufacturer’s protocol. The TPHA test is an indirect hemagglutination test for the determination of antibodies specific to *Treponema pallidum* which is a common confirmatory test in many algorithms for syphilis testing [30].

### Cervical swabs

Cervical cells were collected using the CareBrush® (QIAGEN, Gaithersburg, MD, USA) after inserting the right-sized speculum into the vagina and focusing the cervix. CareBrush® is a cytology sampling device manufactured by QIAGEN, Gaithersburg, MD, USA and was approved by US Food and Drug Administration (FDA) to be used for cervical sample collection. After cervical sampling, the brush heads were transferred directly into a vial containing the alcohol-based preservative careHPV collection medium (CareHPV® collection medium DCM; QIAGEN, Gaithersburg, MD, USA), for storage and transportation to the laboratory. CareBrush® and CareHPV® collection medium (DCM) have been used for cervical sampling and storage in many previous studies in Uganda [31–32].

While in the field, the vial containing the cervical samples were stored and transferred into a liquid nitrogen tank for shipment to immunology laboratory in Makerere University College of Health Sciences, Kampala, Uganda. All samples were shipped from the field to the laboratory every fourth night.

### CLART HPV2 testing

Cervical swabs were analyzed with a HPV genotyping test, CLART^®^ HPV2 (Genomica, Madrid, Spain) which is based on PCR followed by microarray for determination of genotype. CLART HPV2 (Genomica, Madrid Spain) is a low-density microarray assay based on PCR amplification of genotype specific HPV L1 fragments from 35 individual HPV genotypes (6, 11, 16, 18, 26, 31, 33, 35, 39, 40, 42, 43, 44, 45, 51, 52, 53, 54, 56, 58, 59, 61, 62, 66, 68, 70, 71, 72, 73, 81, 82, 83, 84, 85 and 89), with analytical sensitivity calibrated against known copies of cloned plasmids.

Two hundred and fifty (250) μL of the diluted careHPV sample was spun down (15 min, 14,000 revolutions per minute), with the supernatant removed and cell pellet re-suspended and incubated with proteinase K for two hours at 56°C. HPV DNA was purified using QIACube with the QIAcube mini kit according to instructions by the manufacturer (QIAGEN).

Five (5) μl of purified DNA were used for the PCR amplification and 50 μl PCR-reactions were run on PCR-equipment EppendorfMastercycler ep gradient S (Eppendorg AG, Hamburg, Germany). Each reaction contained 45 μl reaction mix (Genomica, Madrid Spain), 0.75 mM MgCl (Thermo Fisher Scientific, Waltham, USA) together with sample DNA. PCR-program included an initial denaturation step at 95° for 5 minutes followed by 40 cycles of 95° at 30 seconds, 55° for 60 seconds and 72° for 90 seconds. PCR-program ended with a 4° step for 8 minutes. Visualization was performed on the CLART microarray, using 5 μl of the denatured PCR products. Hybridization between the amplicons and their specific probes on CLART results in formation of an insoluble precipitate of peroxidase when adding a Streptavidin conjugate that bind to the biotin-labeled PCR products. Precipitate is analyzed on the Clinical Array Reader (Genomica, Madrid, Spain) with CLART^®^ Human papillomavirus 2 software. All samples returning an invalid outcome were retested, and the second result was considered definitive.

CLART HPV2 assay was chosen for HPV genotyping in this study because it has demonstrated performance comparable to Hybrid Capture 2 (HC2) in terms of concordance level, clinical sensitivity and specificity. The agreement between CLART HPV2 and HC2 were both very good concordance levels in the ranges of 98.6–98.8% respectively [33]. The clinical sensitivity of CLART HPV2 against CIN2+ were in the ranges of 96–96.9% which were comparable with HC2 with 71.4% sensitivity [24]. The specificity of CLART HPV2 against CIN2+ were in the ranges of 71.9–73.6% [31]. Similarly, CLART HPV2 has demonstrated performance comparable to Linear Array. The agreement between CLART HPV 2 and Linear Array was 88.7% concordance level [34]. The clinical sensitivity of CLART HPV2 by positive predictive value of CIN2+ in ASCUS were 67.3% vs 57.1%) [35].

### Data analysis

Data from the laboratory tests and questionnaire were coded, entered and analyzed with Statistical Package for Social Sciences (SPSS) version 22.0 for windows. The primary outcome variable for this study was the binary (positive or negative) HPV test result which was further categorized into any HPV, vaccine HPV-16/18 and non-vaccine HPV strains. The study arms i.e. HPV-16/18-vaccinated and non-vaccinated groups were compared on categorical variables using Chi square statistics or Fisher’s exact tests when frequency of occurrence of the event or status were small, for example <5 [26]. To adjust for factors such as age and age at sexual debut differences between the study groups that have the potential to impact on HPV prevalence, log-binomial regression was performed. On continuous numerical variables, the study groups were compared using independent samples T-test [26]. Hierarchical logistic regression [26] were performed after controlling for participant’s age, age at sexual debut and educational level to identify useful predictors for any HPV, vaccine HPV-16/18 and non-vaccine HPV strains. The level of statistical significance was set at 0.05, two tailed test and 95% confidence intervals were computed where necessary.

## Results

The number of participants eligible for the study was 488, those with HIV test results were 412, those with CD4 count results were 410, those with TPHA (syphilis) test results were 418 and HPV test results were 401 (Fig 1). The age range of the 488 participants eligible for the study which comprised of 51.6% vaccinated young women was 15–24 years and mean age was 18.5 (standard deviation 1.4). Non-eligibility rate was 63.5% and the major reason for non-eligibility was sexual inactivity (i.e. virginity).

### Characteristics of the study participants

The participants’ age range was 15–24 years. The HPV vaccinated group was significantly younger and also initiated sexual activity at significantly younger age than their non-vaccinated counterparts (Table 2). Notably, the differences in age and age at sexual debut between the vaccinated and unvaccinated groups were just means of 8 and 7 months respectively. Otherwise, there were no significant differences between the vaccinated and non-vaccinated groups regarding the demographic and sexual behavioral characteristics (Table 2). With exception of age at sexual debut which was significantly lower among the HPV vaccinated group than the non-vaccinated group, there were no significant differences between the two groups regarding sexual behaviors and BMI (Table 2). Similarly, there was no significant difference between the two groups regarding condom use (39.9% vs 42.0%, p = 0.747).

**Table 2:** Comparison of the HPV vaccinated and non-vaccinated groups by demographic characteristics, STI/HIV status, BMI and sexual behaviors.

Demographic characteristics	Vaccinated group	Non-vaccinated group	*p value*
	**[f (%)]**	**[f (%)]**	
Age group			
**15–19 years**	**217 (86.1)**	**145 (61.4)**	<0.001*
20–24 years	35 (13.9)	91 (38.6)	
Address			
Urban	169 (67.1)	169 (71.6)	0.322
Rural	83 (32.9)	67 (28.4)	
Education level			
**Senior 1–4 class**	**251 (99.6)**	**100 (43.1)**	<0.001*
Senior 5–6 class	1 (0.4)	132 (56.9)	
Other STIs (HIV and Syphilis)			
Positive	3 (1.4)	9 (4.4)	0.121
Negative	209 (98.6)	196 (95.6)	
CD4 count			
<500 cells/μL	2 (38.6)	5 (71.4)	0.276
≥500 cells/μL	207 (51.4)	196 (48.6)	
**Mean age in years at sexual debut (SD)**	**15.6 (2.7)**	**16.2 (3.1)**	0.028*
Mean number of sexual partners past 3 months (SD)	0.56 (0.50)	0.55 (0.60)	0.734
Mean number of sexual partners past 12 months (SD)	0.80 (0.53)	0.84 (0.81)	0.488
Mean number of sexual partners in a lifetime (SD)	1.28 (0.89)	1.24 (0.63)	0.608
Mean body mass index (SD)	22.8 (2.7)	23.0 (3.0)	0.362

f is frequency, % is percentage, STIs is sexually transmitted infections, SD is standard deviation, p value is the level of significance

* Statistically significant p–value.

### HPV genoprevalence

Out of the 401 cervical samples tested, there were 135 HPV positive cases giving an HPV genoprevalence of 33.7% (95% CI 29.1–38.3). The non-vaccine HPV strains were generally more prevalent than the vaccine HPV-16/18 strains (32.4% 95% CI 27.8–37.0 vs. 3.0% 95% CI 1.3–4.7). After adjusting for age and age at sexual debut in log-binomial regression, both models for any HPV prevalence and vaccine HPV prevalence were not statistical significant improvements from one without such factors (Omnibus test likelihood ratio chi square 0.534, df 2, p 0.766 and 2.402, df 2, p 0.301 respectively).

Table 3 shows that the most prevalent non-vaccine HPV strains overall were HPV58, 52, 6, 66 and 51. Among the bivalent HPV16/18-vaccinated group, the most prevalent non-vaccine HPV strains were HPV6, 58, 52, 59 and 51 while among the non-vaccinated group, the most prevalent strains were HPV58, 52, 66, 83 and 51.

**Table 3:** Prevalence of non-vaccine HPV strains among 15–24 year old bivalent HPV vaccinated and non-vaccinated young women in Uganda by 2014.

	All	Vaccinated group	Non-vaccinated group
	(n = 401)	(n = 205)	(n = 196)
HPV type	HPV DNA+ (%)	HPV DNA+ (%)	HPV DNA+ (%)
CARCINOGENIC GROUP 1 OR HIGH RISK TYPES			
HPV16	8 (2.0)	0	8 (4.1)
HPV18	4 (1.0)	1 (0.5)	3 (1.5)
HPV31	7 (1.7)	2 (1.0)	5 (2.6)
HPV33	1 (0.2)	1 (0.5)	0 (0.0)
HPV35	5 (1.2)	1 (0.5)	4 (2.1)
HPV39	5 (1.2)	3 (1.5)	2 (1.0)
HPV45	2 (0.5)	1 (0.5)	1 (0.5)
HPV51	13 (3.2)	5 (2.4)	8 (4.1)
HPV52	19 (4.7)	8 (3.9)	11 (5.6)
HPV56	5 (1.2)	1 (0.5)	4 (2.1)
HPV58	22 (5.2)	8 (3.9)	14 (7.2)
HPV59	12 (3.0)	8 (3.9)	4 (2.1)
PROBABLY CARCINOGENIC (GROUP 2A) TYPES			
HPV68	4 (1.0)	1 (0.5)	3 (1.5)
POSSIBLY CARCINOGENIC (GROUP 2B) TYPES			
HPV66	14 (3.5)	5 (2.4)	9 (4.6)
HPV26	1 (0.2)	0 (0.0)	1 (0.5)
HPV53	6 (1.5)	4 (1.9)	2 (1.0)
HPV70	8 (2.0)	4 (1.9)	4 (2.1)
HPV82	8 (2.0)	3 (1.5)	5 (2.6)
HPV85	0 (0.0)	0 (0.0)	0 (0.0)
LOW RISK (GROUP 3) TYPES			
HPV6	17 (4.2)	12 (5.8)	5 (2.6)
HPV11	5 (1.2)	3 (1.5)	2 (1.0)
HPV40	1 (0.2)	0 (0.0)	1 (0.5)
HPV44	3 (0.7)	1 (0.5)	2 (1.0)
HPV61	8 (2.0)	5 (2.4)	3 (1.5)
HPV62	8 (2.0)	5 (2.4)	3 (1.5)
HPV72	0 (0.0)	0 (0.0)	0 (0.0)
HPV81	4 (1.0)	1 (0.5)	3 (1.5)
HPV83	9 (2.2)	3 (1.5)	6 (3.1)
HPV84	6 (1.5)	2 (1.0)	4 (2.1)
HPV89	1 (0.2)	0 (0.0)	1 (0.5)
NOT CLASSIFIED TYPES			
HPV42	2 (0.5)	2 (1.0)	0 (0.0)
HPV43	0 (0.0)	0 (0.0)	0 (0.0)
HPV54	3 (0.7)	2 (1.0)	1 (0.5)
HPV71	1 (0.2)	1 (0.5)	0 (0.0)
HPV73	0 (0.0)	0 (0.0)	0 (0.0)

HPV+ frequency, % percent. HPV type classification is based on IARC (2012) monograph volume 100B [7]. HPV types included are those included in CLART HPV2 assay and those missing were those that are not part of the CLART HPV2 assay. Not classified types are those HPV genotypes that were not indicated in IARC2012 monograph volume 100b to belong to neither Group 1 nor Group 2A nor Group 2B nor Group 3.

Regardless of the HPV vaccination status, the vaccine HPV-16/18 strains were significantly more prevalent among young women of advanced secondary education, those who tested positive for other STIs (HIV and syphilis) and those with CD4 count <500 cells/ μL. As for the non-vaccine HPV strains, the prevalence were significantly higher among young women from urban address and those who tested positive for other STIs (HIV and syphilis).

#### HPV genoprevalence by bivalent HPV vaccination status

**Fig 2:**
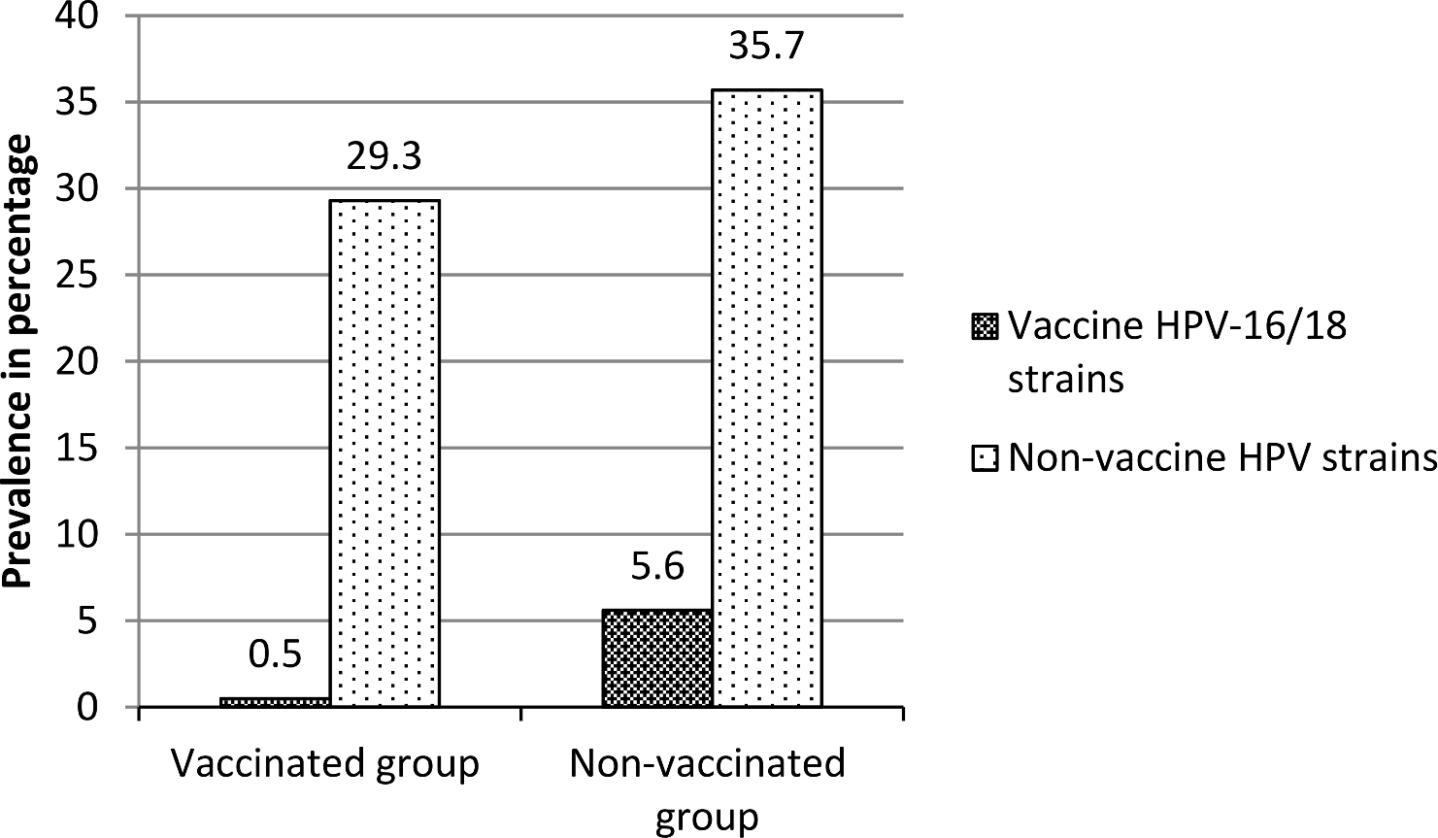
HPV genoprevalence among 15–24 year old non-vaccinated and bivalent HPV vaccinated young women in Uganda by 2014. The vaccinated group comprised of women who received the bivalent HPV 16/18 vaccine whereas the non-vaccinated group did not.

Generally, the genoprevalence of vaccine HPV-16/18 strains was significantly lower among the bivalent HPV16/18-vaccinated group compared to the non-vaccinated group (p 0.006) (Fig 2). As for non-vaccine HPV strains, there were no statistically significant difference between the vaccinated and non-vaccinated groups regarding the prevalence (p 0.203). After adjusting for age, age at sexual debut and bivalent HPV vaccination status in log-binomial regression, vaccine HPV prevalence ratio was statistical significant improvement from one without such factors (Omnibus test likelihood ratio chi square 16.994, df 3, p 0.001). The test of model effect further showed a statistically significant association between bivalent HPV vaccination status and vaccine HPV prevalence (Wald Chi square 3120.619, df 1, p 0.000). Bivalent HPV vaccination decreased the likelihood of vaccine HPV infection (B -19.480, 95% CI -20.163 to– 18.796).

### Predictors for vaccine and non-vaccine HPV DNA positivity

Having a bivalent HPV vaccination status was a protective factor for vaccine HPV-16/18 type positivity, while having another STI (HIV/syphilis) was a risk factor (Table 4). The factors adjusted for in Table 4 were age, age at sexual debut and educational level. As for non-vaccine HPV type positivity, the risk factors were having an urban address, low BMI, multiple sexual partners in a lifetime, other STI and low CD4 count (Table 4).

**Table 4:** Predictors for vaccine and non-vaccine HPV infections.

	Vaccine HPV-16/18 infections		Non-vaccine HPV infections	
Predictors	AOR (95% CI)	*p value*	AOR (95% CI)	*p value*
Bivalent HPV-16/18 vaccination				
Vaccinated	0.08 (0.01–0.64)	0.006*	0.79 (0.46–1.34)	0.377
Non-vaccinated	Ref.			
Address				
Urban area	2.23 (0.30–34.18)	0.331	4.45 (2.23–8.85)	0.000*
Rural area	Ref.			
BMI (kg/m^2^)	0.93 (0.71–1.21)	0.566	0.85 (0.77–0.94)	0.001*
Number of sex partners past 3 months	3.03 (0.58–15.91)	0.190	1.61 (0.94–2.75)	0.082
Number of sex partners past 12 months	0.72 (0.19–2.76)	0.629	1.22 (0.74–2.01)	0.432
Number of sex partners in a lifetime	0.33 (0.04–3.07)	0.328	1.77 (1.17–2.69)	0.007*
Condom use				
Ever used	4.52 (0.86–23.84)	0.075	0.79 (0.46–1.34)	0.377
Never used	Ref.			
Other STIs (HIV/Syphilis)				
Positive	14.21(1.96–102.91)	0.009*	10.83 (2.21–53.08)	0.003*
Negative	Ref.			
CD4 count	1.00 (1.00–1.00)	0.879	1.00 (1.00–1.00)	0.005*

Factors adjusted for were age; age at sexual debut and educational level; AOR is adjusted Odd Ratio

* statistically significant p-value.

## Discussion

Here we report a follow-up study on protective effect of a bivalent HPV16/18 vaccine performed in a group of women aged 15–24 years in Ibanda District, Uganda. The overall 33.7% HPV genoprevalence found in the current study is much lower than the 74.8% from a previous study among Ugandan women of the same age bracket [8]. The difference could be because the current study comprised of 51.6% bivalent HPV vaccinated group while the previous study did not. However, the HPV genoprevalence in the non-vaccinated group was just above 40%, still much lower than the genoprevalence found in previous studies among non-vaccinated young women in Uganda [13, 36].

Our study finding that the genoprevalence of vaccine HPV 16/18 strains combined was significantly lower among the vaccinated group compared to the non-vaccinated group concurs with a number of previous bivalent HPV-16/18 vaccine follow up studies in Germany [21], Scotland [22] and England [23]. These findings also concur with findings from bivalent HPV-16/18 vaccine efficacy trials in Costa Rica [17–18] and Finland [19]. The consistency of the current study finding with previous bivalent HPV16/18 vaccine follow up and vaccine efficacy studies from other parts of the world implies that the bivalent HPV16/18 vaccine is protective against vaccine HPV-16/18 strains in African young women in developing country Uganda and would be useful for primary prevention of cervical cancer.

Our study findings that the genoprevalence of vaccine HPV-16/18 strains were not significantly lower among subgroups of vaccinated girls such as those with other STIs (HIV/syphilis), CD4 count <500 cells/mm^3^, abnormal BMI, multiple lifetime sexual partners and non-condom use when compared to their non-vaccinated counterparts is unique. However, such detailed analysis might be hampered by lack of power in our study. In contrast, the findings from multivariate analysis of our study revealed that a significant risk factor for vaccine HPV-16/18 type positivity was other STIs (HIV/syphilis) as opposed to bivalent HPV vaccination which was a protective factor. As for non-vaccine HPV type positivity, other STI plus women’s number of sexual partners, underweight (BMI<18.5 kg/m^2^) and low CD4 count <500 cells/mm^3^ were risk factors. The association between other STI, particularly HIV infection, and HPV positivity observed in our study is consistent previous HPV studies in Uganda [13, 36] and also consistent with a HPV vaccine follow up study in Germany [26] were they also identified other STIs particularly HIV as a useful predictor of HPV positivity in multivariate models that factored HPV vaccination status among the potential predictors. As earlier noted, this finding may also imply that catch up or supplementary HPV vaccination campaigns should be possibly targeted at the aforementioned subgroups of girls at an increased risk of HPV infection. The findings though points to the need for simultaneous use and integration of interventions known to be effective against HIV/syphilis, malnutrition and sexual risk behaviors into existing HPV vaccination programme targeted at young girls in countries in Africa such as Uganda.

The strengths of the current study included inclusion of sexually active young women as confirmed by verbal interview, inclusion of bivalent HPV fully vaccinated group whose vaccination status was first obtained verbally and then confirmed by HPV vaccination register, inclusion of a non-vaccinated comparison group that was statistically not any different from the vaccinated group on key predictors of HPV infections, use of a broad spectrum HPV DNA genotyping assay (CLART, Genomica, Madrid, Spain) that covers 35 HPV genotypes [30] and laboratory testing of other sexually transmitted infections (HIV and syphilis). On the other hand, the use of cross sectional design to compare the Bivalent HPV16/18 vaccinated and non-vaccinated women regarding HPV prevalence could not allow for rigorous determination of cause-effect relationship between Bivalent HPV16/18 vaccination and HPV infection. Also, the lack of anti-HPV antibodies data could not allow for assessment of concordance or discordance between HPV infection status and anti-HPV antibody levels. Therefore, longitudinal study designs where e.g. study of participants whose HPV status is known from baseline, and corroboration of anti-HPV antibody data with HPV infection status are recommended for future studies. Whereas the powering of the study to detect a 10-fold reduction in vaccine HPV 16/18 strains in the vaccinated compared to the unvaccinated group was based on the vaccine efficacy data from clinical trials [18–21], this has consequently led to under powering of the study for subgroup analyses and other interesting comparisons e.g. by individual genotype. It should however be noted that subgroup analyses and comparison by individual genotypes were not among the primary objectives of the study and therefore caution should be taken in declaring no difference based on statistical tests of this study.

It can be argued that the fact that the vaccinated group were younger and initiated sexual activity earlier than the unvaccinated group, they had prolonged exposure and the opportunity to develop persistent HPV infection. These may make age and age at sexual debut important confounders to the association between HPV vaccination status and HPV infection. It should however be noted that the differences in age and age at sexual debut between the vaccinated and unvaccinated group were means of 8 and 7 months respectively, which were too short a time for differential HPV exposure nor persistence. A previous prospective cohort study of Ugandan young women of the same age group (12–24 years) found neither incident HPV infection nor HPV clearance were associated with age after as long as 18.5 months and 226 person-years of follow up [15]. Nevertheless, to account for age and age at sexual debut difference between vaccinated and unvaccinated groups and their unlikely impact on HPV acquisition or clearance, we further adjusted for age and age sexual debut in log-binomial regression for prevalence ratio and logistic regression for predictors of HPV infection (Table 4).

## Conclusions

We found a significantly lower genoprevalence of vaccine HPV-16/18 strains among the bivalent HPV-16/18 vaccinated (Cervarix®) young women compared to their non-vaccinated counterparts in Uganda 5.5 years after bivalent HPV-16/18 vaccine implementation. Other STIs (HIV/syphilis) persists to be a risk factor for vaccine HPV-16/18 infection despite bivalent HPV vaccination. Our data concurs with the findings of bivalent HPV-16/18 vaccine efficacy trials and other previous bivalent HPV vaccine follow up studies from other parts of the world. This study reinforces the recommendation to vaccinate young girls in adolescence before sexual debut and the need to integrate other sexual health services into HPV vaccination programme targeted at young girls.

## References

[1] International Agency for Research on Cancer (IARC) and WHO: Globocan 2012: Estimated cancer incidence, mortality and prevalence worldwide in 2012. Available at http://globocan.iarc.fr/pages/fact_sheet_population.

[2] Wabinga HR, Nambooze S, Amulen PM, Okello C, Mbus L, Parkin DM. Trends in the incidence of cancer in Kampala, Uganda 1991–2010. Int J Cancer. 2014 7 15; 135(2):432–9. 10.1002/ijc.28661 24615279

[3] Walboomers JM, Jacobs MV, Manos MM, Bosch FX, Kummer JA, Shah KV, Snijders PJ, Peto J, Meijer CJ, Muñoz N. Human papillomavirus is a necessary cause of invasive cervical cancer worldwide. J Pathol. 1999; 189(1): 12–9. 10451482 10.1002/(SICI)1096-9896(199909)189:1<12::AID-PATH431>3.0.CO;2-F

[4] Munoz N, Castellsague X, de Gonzalez AB, Gissmann L. Chapter 1: HPV in the etiology of human cancer. Vaccine 2006; 24S3: S1–S10. 10.1016/j.vaccine.2006.05.115 16949995

[5] Muñoz N, Bosch FX, de Sanjosé S, Herrero R, Castellsagué X, Shah KV, et al International Agency for Research on Cancer Multicenter Cervical Cancer Study Group. Epidemiologic classification of human papillomavirus types associated with cervical cancer. N Engl J Med. 2003; 348(6): 518–27. 12571259 10.1056/NEJMoa021641

[6] Schiffman M, Clifford G, Buonaguro FM. Classification of weakly carcinogenic human papillomavirus types: addressing the limits of epidemiology at the borderline. Infect. Agent Cancer 2009; 4: 8 10.1186/1750-9378-4-8 19486508 PMC2694995

[7] International Agency for Research on Cancer (IARC). Monographs on the Evaluation of Carcinogenic Risks to Humans Volume 100B: A Review of Human Carcinogens: Biological Agents. Lyon: International Agency for Research on Cancer 2012.

[8] Bzhalava D, Guan P, Franceschi S, Dillner J, Clifford G. A systematic review of the prevalence of mucosal and cutaneous human papillomavirus types. Virology 2013; 445: 224–231. 10.1016/j.virol.2013.07.015 23928291

[9] Bernard HU, Calleja-Macias IE, Dunn ST. Genome variation of human papillomavirus types: phylogenetic and medical implications. Int J Cancer. 2006; 118(5): 1071–6. 16331617 10.1002/ijc.21655

[10] Bernard HU, Burk RD, Chen Z, van Doorslaer K, Hausen H, de Villiers EM. Classification of papillomaviruses (PVs) based on 189 PV types and proposal of taxonomic amendments. Virology 2010; 401: 70–79. 10.1016/j.virol.2010.02.002 20206957 PMC3400342

[11] Odida M, de Sanjosé S, Quint W, Bosch FX, Klaustermeier J, Weiderpass E. Human Papillomavirus type distribution in invasive cervical cancer in Uganda. BMC Infect Dis 2008; 8: 85 10.1186/1471-2334-8-85 18577214 PMC2459185

[12] Banura C, Mirembe FM, Katahoire AR, Namujju PB, Mbonye AK, Wabwire FM. Epidemiology of HPV genotypes in Uganda and the role of the current preventive vaccines: A systematic review. Infect Agent Cancer 2011; 6: 11 10.1186/1750-9378-6-11 21749691 PMC3163594

[13] Banura C, Franceschi S, Doorn LJ, Arslan A, Wabwire-Mangen F, Mbidde EK, et al Infection with human papillomavirus and HIV among young women in Kampala, Uganda. J Infect Dis 2008; 197(4): 555–62. 10.1086/526792 18237268

[14] Odida M, Sandin S, Mirembe F, Kleter B, Quint W, Weiderpass E. HPV types, HIV and invasive cervical carcinoma risk in Kampala, Uganda: a case-control study. Infectious Agents and Cancer 2011, 6:8 doi: http://www.infectagentscancer.com/content/6/1/8 21702999 10.1186/1750-9378-6-8 PMC3141535

[15] Banura C, Sandin S, van Doorn L, Quint W, Kleter B, Wabwire-Mangen F, Mbidde EK and Weiderpass E. Type-specific incidence, clearance and predictors of cervical human papillomavirus infections (HPV) among young women: a prospective study in Uganda. Infectious Agents and Cancer 2010, 5:7 10.1186/1750-9378-5-7 20380709 PMC2873244

[16] Banura C, Franceschi S, van Doorn LJ, Arslan A, Kleter B, Wabwire-Mangen F, Mbidde EK, Quint W, Weiderpass E. Prevalence, incidence and clearance of human papillomavirus infection among young primiparous pregnant women in Kampala, Uganda. Int J Cancer. 2008; 123(9): 2180–7. 10.1002/ijc.23762 18711697

[17] LaMontagne DS, Barge S, Le NT, Mugisha E, Penny ME, Gandhi S, et al Human papillomavirus vaccine delivery strategies that achieved high coverage in low-and middle income countries. Bull World Health Organ 2011; 89: 821–830B. 10.2471/BLT.11.089862 22084528 PMC3209730

[18] Paavonen J, Naud P, Salmeron J, Wheeler CM, Chow SN, Apter D, et al Efficacy of human papillomavirus (HPV)-16/18 AS04-adjuvanted vaccine against cervical infection and precancer caused by oncogenic HPV types (PATRICIA): final analysis of a double-blind, randomised study in young women. Lancet 2009; 374(9686): 301–14. 10.1016/S0140-6736(09)61248-4 19586656

[19] Hildesheim A, Wacholder S, Catteau G, Struyf F, Dubin G, Herrero R, et al Efficacy of the HPV-16/18 vaccine: final according to protocol results from the blinded phase of the randomized Costa Rica HPV-16/18 vaccine trial. Vaccine 2014 9 3; 32(39): 5087–97. 10.1016/j.vaccine.2014.06.038 Epub 2014 Jul 10. 25018097 PMC4166498

[20] Palmroth J, Merikukka M, Paavonen J, Apter D, Eriksson T, Natunen K, et al Occurrence of vaccine and non-vaccine human papillomavirus types in adolescent Finnish females 4 years post-vaccination. Int. J. Cancer 2013; 131: 2832–2838. 10.1002/ijc.27586 22492244

[21] Lu B, Kumar A, Castellsagué X, Giuliano AR. Efficacy and Safety of Prophylactic Vaccines against Cervical HPV Infection and Diseases among Women: A Systematic Review & Meta-Analysis. BMC Infectious Diseases 2011; 11: 13 doi: http://www.biomedcentral.com/1471-2334/11/13 21226933 10.1186/1471-2334-11-13 PMC3034689

[22] Delerél Y, Remschmidt C, Leuschner J, Schuster M, Fesenfeld M, Schneider A, et al Human Papillomavirus prevalence and probable first effects of vaccination in 20 to 25 year-old women in Germany: a population-based cross sectional study via home-based self-sampling. BMC Infectious Diseases 2014; 14:87 10.1186/1471-2334-14-87 24552260 PMC3933406

[23] Kavanagh K, Pollock KG, Potts A, Love J, Cuschieri K, Cubie H, et al Introduction and sustained high coverage of the HPV Bivalent vaccine leads to a reduction in prevalence of HPV 16/18 and closely related HPV types. Br J Cancer 2014; 110(11): 2804–11. 10.1038/bjc.2014.198 24736582 PMC4037824

[24] Mesher D, Soldana K, Howell-Jonesa R, Panwarb K, Manyengab P, Jit M, et al Reduction in HPV 16/18 prevalence in sexually active young women following the introduction of HPV immunization in England. Vaccine 2014; 32: 26–32. 10.1016/j.vaccine.2013.10.085 PMC3898718 24211166

[25] Polit F. P., & Beck C. T. Nursing research: Generating and assessing evidence for nursing practice 2008 Philadelphia, PA: Lippincott, Williams, & Wilkins.

[26] Altman D. G. Practical statistics for medical research 1991 London: Chapman & Hall.

[27] World Health Organization. Physical status: the use and interpretation of anthropometry: Report of a WHO Expert Committee. World Health Organ Tech Rep Ser 1995; 854: 1–452. 8594834

[28] Downing RG, Otten RA, Marum E, Biryahwaho B, Alwano-Edyegu MG, Sempala SD, et al Optimizing the delivery of HIV counseling and testing services: the Uganda experience using rapid HIV antibody test algorithms. J Acquir Immune Defic Syndr Hum Retrovirol 1998 8 1; 18(4): 384–8. 9704945 10.1097/00042560-199808010-00011

[29] Johnson D, Hirschkorn D, Busch MP. Evaluation of four alternative methods for determination of absolute CD4+ lymphocyte counts. The National Heart, Lung and Blood Institute Retrovirus Epidemiology Donor Study. J. Acquir. Immune Defic. Syndr. Hum. Retrovirol 1995; 15: 522–530. 8548331

[30] Luger A, Schmidt BL, Gschnait F. Recent progress in syphilis serology. Wien Klin Wochenschr 1983; 95(13): 4 40–3. 6356627

[31] Jeronimo J, Bansil P, Lim J, Peck R, Paul P, Amador JJ, et al A multicountry evaluation of careHPV testing, visual inspection with acetic acid, and papanicolaou testing for the detection of cervical cancer. Int J Gynecol Cancer 2014 3; 24(3):576–85. 10.1097/IGC.0000000000000084 24557438 PMC4047307

[32] Moses E, Pedersen HN, Mitchell SM, Sekikubo M, Mwesigwa D, Singer J, et al Uptake of community-based, self-collected HPV testing vs. visual inspection with acetic acid for cervical cancer screening in Kampala, Uganda: preliminary results of a randomised controlled trial. Trop Med Int Health. 2015 10;20(10):1355–67. 10.1111/tmi.12549 Epub 2015 Jun 28. 26031572

[33] Pista A, Verdasca N, Oliveira A. Clinical performance of the CLART human papillomavirus 2 assay compared with the hybrid capture 2 test. J Med Virol 2011; 83(2): 272–6. 10.1002/jmv.21952 21181922

[34] Chranioti A, Spathis A, Aga E, Meristoudis C, Pappas A, Panayiotides I, et al Comparison of two commercially available methods for HPV genotyping: CLART HPV2 and Linear Array HPV Genotyping tests. Anal Quant Cytopathol Histpathol 2012; 34(5): 257–63. 23301385

[35] Hubbard RA. Human papillomavirus testing methods. Arch Pathol Lab Med 2003; 127(8): 940–5. 12873165 10.5858/2003-127-940-HPTM

[36] Banura C, Sandin S, van Doorn LJ, Quint W, Kleter B, Wabwire-Mangen F, et al Type-specific incidence, clearance and predictors of cervical human papillomavirus infections (HPV) among young women: a prospective study in Uganda. Infect Agent Cancer 2010; 5: 7 10.1186/1750-9378-5-7 20380709 PMC2873244

